# An Efficient Growth Pattern Algorithm (GrowPAL) for Cluster Structure Prediction

**DOI:** 10.1021/acs.jctc.4c00365

**Published:** 2024-05-31

**Authors:** Carlos López-Castro, Filiberto Ortiz-Chi, Gabriel Merino

**Affiliations:** † Departamento de Física Aplicada, Centro de Investigación y de Estudios Avanzados del Instituto Politécnico Nacional, Mérida 97310, Yucatán, México; ‡ Conahcyt-Departamento de Física Aplicada, Centro de Investigación y de Estudios Avanzados del Instituto Politécnico Nacional, Antigua Carretera a Progreso km 6, Mérida, Yucatán 97310, México

## Abstract

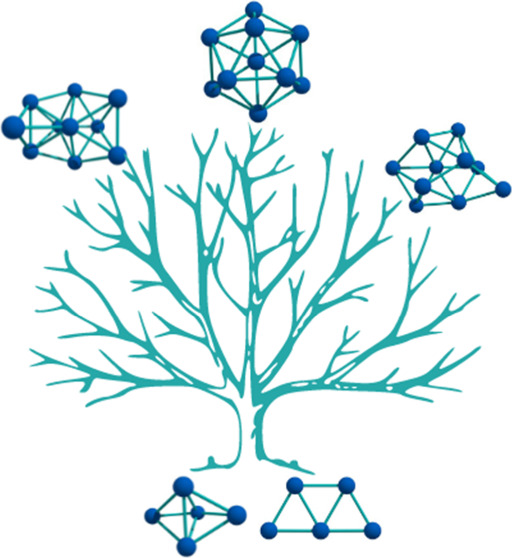

Identifying the lowest energy isomers in large clusters is a major challenge. Here, we introduce the Growth Pattern Algorithm (GrowPAL), a new approach that generates initial seeds composed of *n+1* atoms from the system with *n* atoms through an interstitial-type addition (I-type) mechanism. We evaluated the effectiveness of GrowPAL on Lennard-Jones (LJ) clusters with up to *n* = 80 atoms, verifying the algorithm’s ability to find challenging minima such as LJ_38_ and the partially icosahedral LJ_69_ with fewer optimizations than existing methods. In addition, we discuss the advantages and limitations of GrowPAL using our deconstruction scheme, which identifies “forebears” structures to study growth pathways. Having evaluated the strengths and weaknesses of GrowPAL, we employed it to explore Sutton-Chen clusters containing 5 to 80 atoms, uncovering three new lowest energy forms. We then applied GrowPAL to boron clusters containing 8 to 15 atoms, successfully identifying all reported minima. Overall, GrowPAL offers a practical solution for efficiently identifying global minima in hierarchical systems, thereby reducing computational costs.

## Introduction

Exploring clusters is crucial in chemistry because their properties, which depend heavily on the number of atoms, gradually change as they approach those of bulk materials.^1^ However, finding the lowest energy forms for large clusters is challenging since global optimization algorithms often start with randomly generated seeds, leading to high computational costs and limiting scalability. To address this challenge, various strategies have been developed to simplify the exploration process and save costs. One such strategy is based on transfer learning, which uses knowledge from solved problems to boost performance in systems with a certain degree of homogeneity. Several authors have proposed transfer learning schemes to efficiently explore the potential energy surfaces (PESs) of clusters. For example, Bulusu and co-workers used biased searches and growth schemes based on structural motifs to accelerate the PES exploration of germanium clusters.^2^ Kumar and Kawazoe analyzed the growth pattern of Pt clusters with up to 44 atoms and found that adding a single atom can lead to interesting minima.^3^ Similarly, Nie and co-workers identified growth pathways for Pt clusters through atom addition, identifying reported low-energy forms.^4^ These studies highlighted atom addition as an efficient strategy for exploring energy landscapes and showed that integrating high-quality seeds can significantly reduce the computational cost of complex algorithms.

Lennard-Jones (LJ) clusters have been well-established as popular test cases for various global optimization algorithms due to their hierarchical structure.^5^ These algorithms include basin hopping,^6^ genetic algorithms,^7−11^ simulated annealing,^12^ lattice-based searches followed by local optimization,^13^ hypersurface deformations,^14^ stochastic search,^15^ and particular strategies based on transfer learning. Let us focus on transfer learning. For example, Shao and co-workers introduced a random tunneling algorithm that efficiently explores LJ_*n*_ clusters with *n* up to 330, using seeds from previous scans (LJ_*n-1*_) and decahedral and icosahedral motifs.^16^ It is worth mentioning that the Mackay icosahedron is known to be the dominant motif for LJ clusters ranging from 10 to 150 atoms;^17^ however, the global minimum for some cluster sizes deviates from the icosahedral structure, representing a challenge for global minimization efforts. Wales suggested that identifying the global minimum for LJ_38_ is a crucial benchmark for any global search algorithm while unveiling the decahedron structure of LJ_75_ is considered a “much more severe test” for any algorithm.^6^ LJ_38_ is singular because its global minimum is a face-centered cubic truncated octahedron,^14^ while lower energy decahedral forms characterize the LJ_75–78_ clusters.

**Figure 1:**
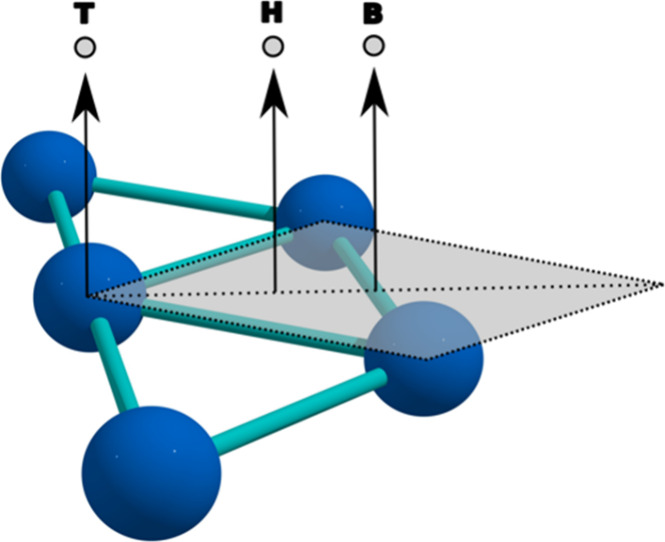
Addition-Types in the Systematic Cluster Growth algorithm. Top (T) addition is performed directly above the selected atom. Bridge (B) addition takes place at the midpoint between two atoms. Hollow addition (H) occurs when an atom is added above the center of the plane formed by three atoms, creating a superficial tetrahedron.

Rodríguez-Kessler et al.^18^ used a different approach called Systematic Cluster Growth (SCG), which generates structures using seeds that may include global minima. Atoms are added at strategic locations within the cluster (top (T), bridge (B), and hollow (H) sites), as shown in Figure 1. These structures are then relaxed and ranked by energy. While the SCG scheme has effectively identified the correct minima for most LJ clusters with fewer than 75 atoms, it does not work for nonicosahedral cases, such as LJ_38_ and LJ_75–78_. This highlights the inherent limitations of T, B, and H-type additions.

Here, we introduce an alternative, the Growth Pattern Algorithm (GrowPAL), which is integrated into the Global Optimization of Molecular Systems (GLOMOS) software.^19^ GrowPAL generates seeds from user-provided structures to make it easier to find large clusters. GrowPAL shows a reduction in the total number of optimizations required compared to previous growth pattern strategies. This efficiency gain is attributed to the novel interstitial addition, which is more effective than prior methods. GrowPAL can also perform parallel local optimizations and structural comparisons, enabling users to choose the number of low-lying energy isomers as seeds for subsequent global optimization stages. We used GrowPAL to explore the PES of LJ_*n*_ clusters ranging from *n* = 5 to 80 atoms as a main test. The growth of each LJ_*n+1*_ is solely determined by the structures identified in the preceding iteration. GrowPAL found the elusive LJ_38_ and LJ_69_, but it was unable to locate LJ_75-78_. The search was initiated solely from the information on LJ_4_ minima.

To further validate the efficacy of GrowPAL, we applied it to the search of clusters described by the Sutton-Chen (SC) family of potentials using the parameters for Cu, Ag, and Au (scalable to Ni, Rh, and Pt, respectively). Furthermore, we applied GrowPAL to the search for boron clusters containing 8 to 15 atoms. The results show that GrowPAL correctly described every minimum, including the tubular structure proposed by Chen and co-workers for B_14_.

## Computational Details

GLOMOS was developed to explore the potential energy surfaces of clusters and molecules by using electronic structure codes to compute the energy as the objective function. GrowPAL, like GLOMOS, is entirely written in Python3, ensuring consistency in modularity, minimal dependencies, and overall philosophy. With the interfaces provided by GLOMOS, GrowPAL can interact with external computational programs like Gaussian 16,^20^ the Vienna *ab initio* Simulation Package (VASP),^21,22^ and the General Utility Lattice Program (GULP),^23^ among others, to perform structural relaxation. Like GLOMOS, GrowPAL’s startup process is meant to be user-friendly and transparent, requiring only the name of the seed file and the type of atom to be added, making the setup process easier for users.

## Algorithm Details

### Interstitial Addition

For a system composed of *n+1* atoms, GrowPAL requires a set of seeds that originate from the system with *n* atoms. These seeds should ideally represent the best solutions for that combination of atoms, namely, the lowest energy isomers for the *n*-system. Such high-quality seeds are crucial, as they enhance the algorithm’s performance.

**Figure 2:**
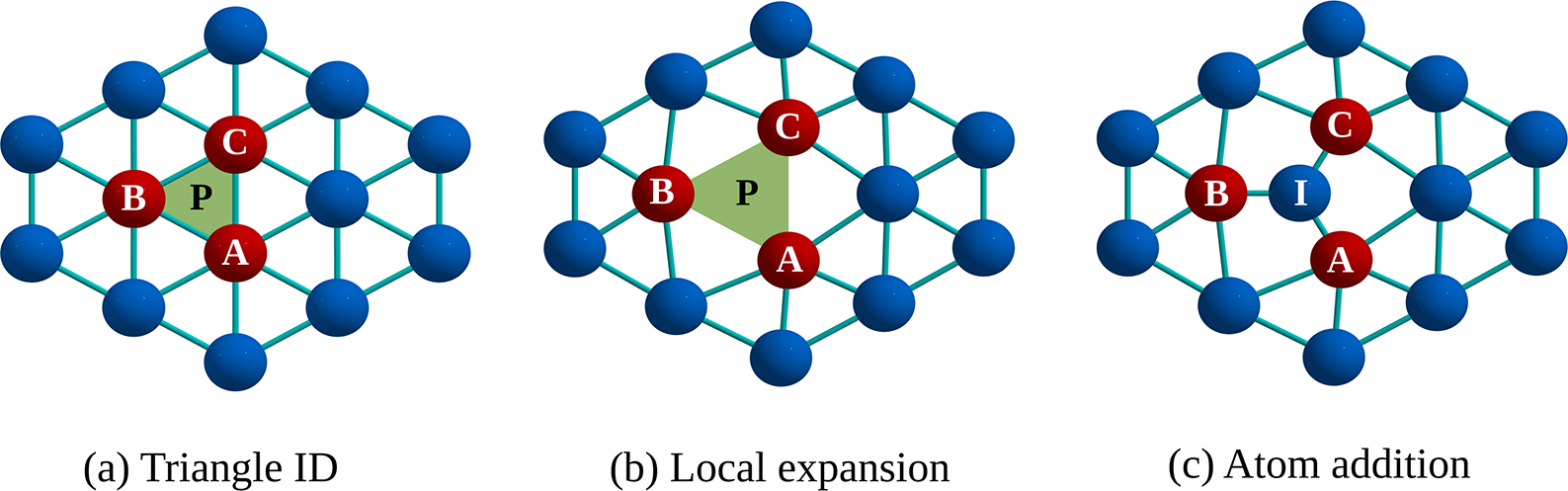
Interstitial Addition. (a) Identification of an inequivalent internal triangle **ABC**, comprising a nonequivalent atom (A) and two neighboring atoms (B and C), and the centroid **P**; (b) Local expansion of the cluster centered around **P**; (c) Addition of a new atom at **P**.

GrowPAL generates new structures from each seed through an interstitial-type addition (I-type) mechanism. This method begins with a detailed symmetry analysis of each seed to facilitate the insertion of an atom into nonequivalent interstitial sites. After selecting a seed, GrowPAL uses the PointGroupAnalyzer class of the Python Materials Genomics (PymatGen) library to identify all nonequivalent atoms within the cluster.^24^ This step is critical for optimizing the generation of new structures, as it reduces redundancy and ensures diversity of configurations. The method then uses each nonequivalent atom and its neighbors to map all the internal triangles. An example of this is depicted in Figure 2a, where the triangle **ABC** is formed by nonequivalent atom **A** and its two neighbors, **B** and **C**.

As depicted in Figure 2b, the cluster undergoes a local expansion centered around triangle ABC’s centroid (P). This expansion is facilitated by scaling the position vector of each atom with the factor *ε* = k*exp(−*r*), where *k* represents the atomic radius and *r* denotes the distance from each atom to **P**. The exponential nature of *ε* ensures that the expansion is concentrated around **P**, effectively preventing overlaps and preserving the structural integrity of the cluster to the greatest extent possible. This process–comprising triangle identification, local expansion, and atom insertion–is repeated for every pair of nonequivalent and neighboring atoms within the cluster. After completing these steps for one seed, GrowPAL moves on to the next seed, applying the same procedure.

It is important to note that I-type addition differs from the hollow-type addition employed by Rodríguez-Kessler and co-workers.^18^ Unlike H-type addition, which is applied to inequivalent triangles on the cluster’s surface, I-type addition introduces a new atom into a section of the cluster that has been locally expanded, facilitating the exploration of structures with internal and external modifications.

### Similarity Discrimination

Although atoms are added with great precision, there is a possibility that different seeds will generate identical trial structures. When these structures are relaxed, they may converge to the same minimum, wasting valuable computational resources. GLOMOS uses the Ultrafast Shape Recognition (USR) algorithm to address this issue.^25^ This algorithm begins by identifying 12 molecular descriptors for each structure, **i** and **j**, to be compared. These descriptors are derived from calculating the first three central moments, which take into account the positions of atoms relative to four reference points: the centroid, the closest atom to the centroid, the furthest atom from the centroid (FCT), and the most distant atom from FCT.

After identifying the descriptors, the similarity coefficient (S_ij_) between the two structures is determined. S_ij_ is calculated as the inverse of the Manhattan distance, which is then transferred and scaled based on the list of descriptors. A similarity coefficient of S_ij_ = 1 indicates maximum similarity, whereas S_ij_ = 0 denotes no similarity. In our study, we set a tolerance threshold of 0.98. To further enhance the speed of comparisons, the origin of the coordinate system for each cluster is shifted to its centroid, reducing the number of required descriptors from 12 to 8.

### Structural Relaxation

All local optimizations were carried out using GULP, which includes Lennard-Jones potential. This protocol begins with a conjugate gradient algorithm, which switches to the Broyden-Fletcher-Goldfarb-Shanno^26^ algorithm once the gradient norm decreases to 0.0001 J.m^–2^. We used a supercell approach to mitigate the effects of periodic boundary conditions, with an 18 Å vacuum between periodic images. The relaxations were performed at a fixed volume. We employed the Lennard-Jones potential with a cutoff range from 0 to 15 Å. The depth of the potential well was set to 1 eV, and the equilibrium distance (2^1/6^σ) was established at 3 Å. The optimization was converged under two conditions: completion of 950 cycles or when the change in energy between iterations fell below a default threshold.
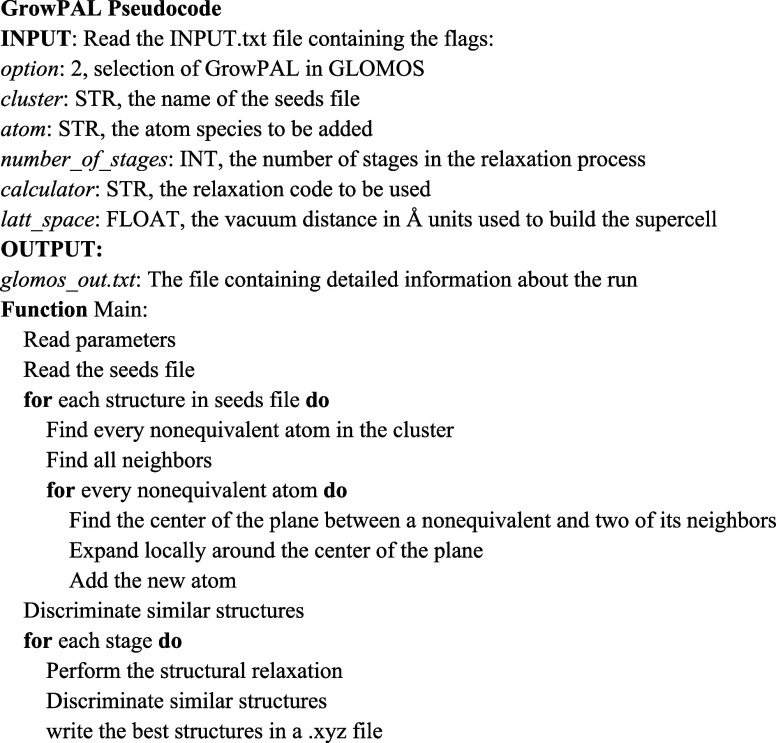


## Results and Discussion

GrowPAL found the global minimum for all LJ clusters up to 74 atoms, including the nonicosahedral LJ_38_ and the partially icosahedral LJ_69_, which has an incomplete core. In addition, the algorithm successfully described clusters with 79 and 80 atoms. A key factor in locating these minima was determining the maximum number of seeds (*N*_*max*_) to consider in each global search. Through rigorous testing, a *N*_*max*_ value of 63 was found to be essential for ensuring the algorithm’s capability to find all global minima from LJ_5_ to LJ_74_. Interestingly, lowering *N*_*max*_ below 63 caused the algorithm to fail to locate the correct global minimum for LJ_38_. Additionally, the exclusive use of I-type addition helped to minimize the total number of optimizations required to under 16,000 while still accurately identifying the correct minima. The energies and the number of optimizations performed to find all global minima up to 74 atoms, with *N*_*max*_ set to 63, are listed in Table 1.

**Table 1:**
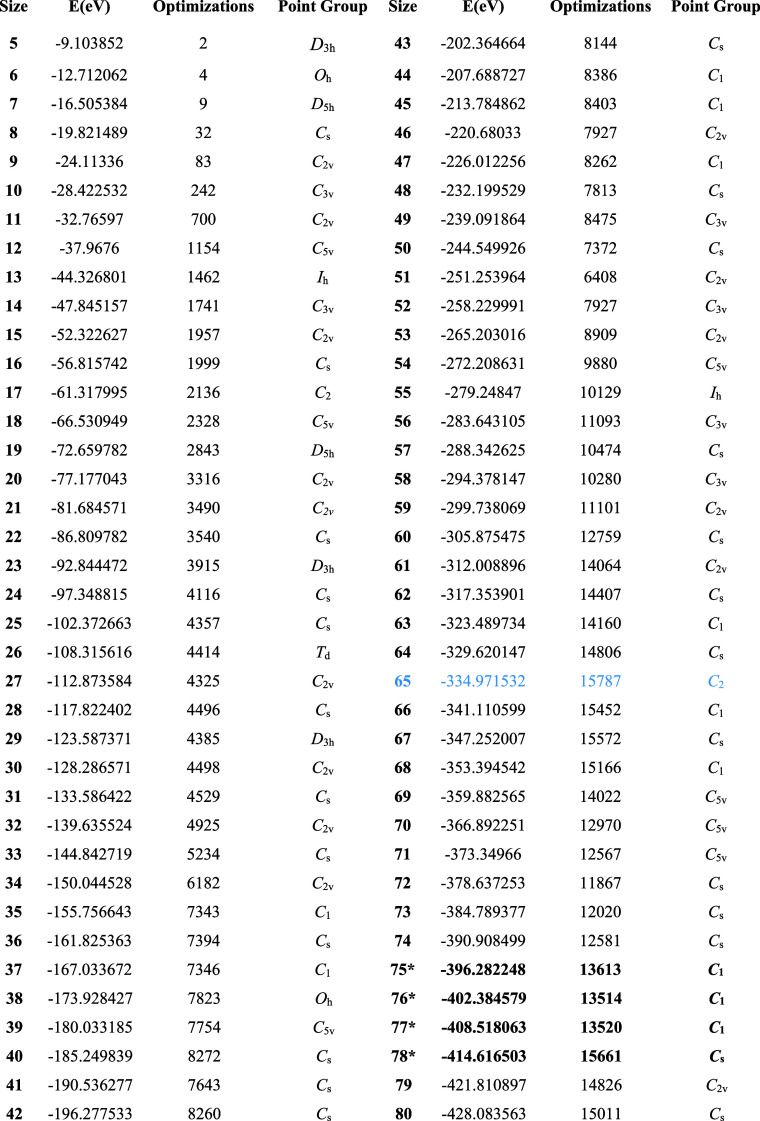
Total Energies and Optimizations Required by GrowPAL to Find Global Minima in LJ Clusters up to 75 atoms, with a Maximum of 63 Seeds Used from Each Preceding Search^a^

a Up to LJ_74_, all energies and symmetries agree with reported values. LJ_65_, requiring the highest number of optimizations, is highlighted in blue. An asterisk (*) next to a result indicates that the global minimum was not found.

**Figure 3:**
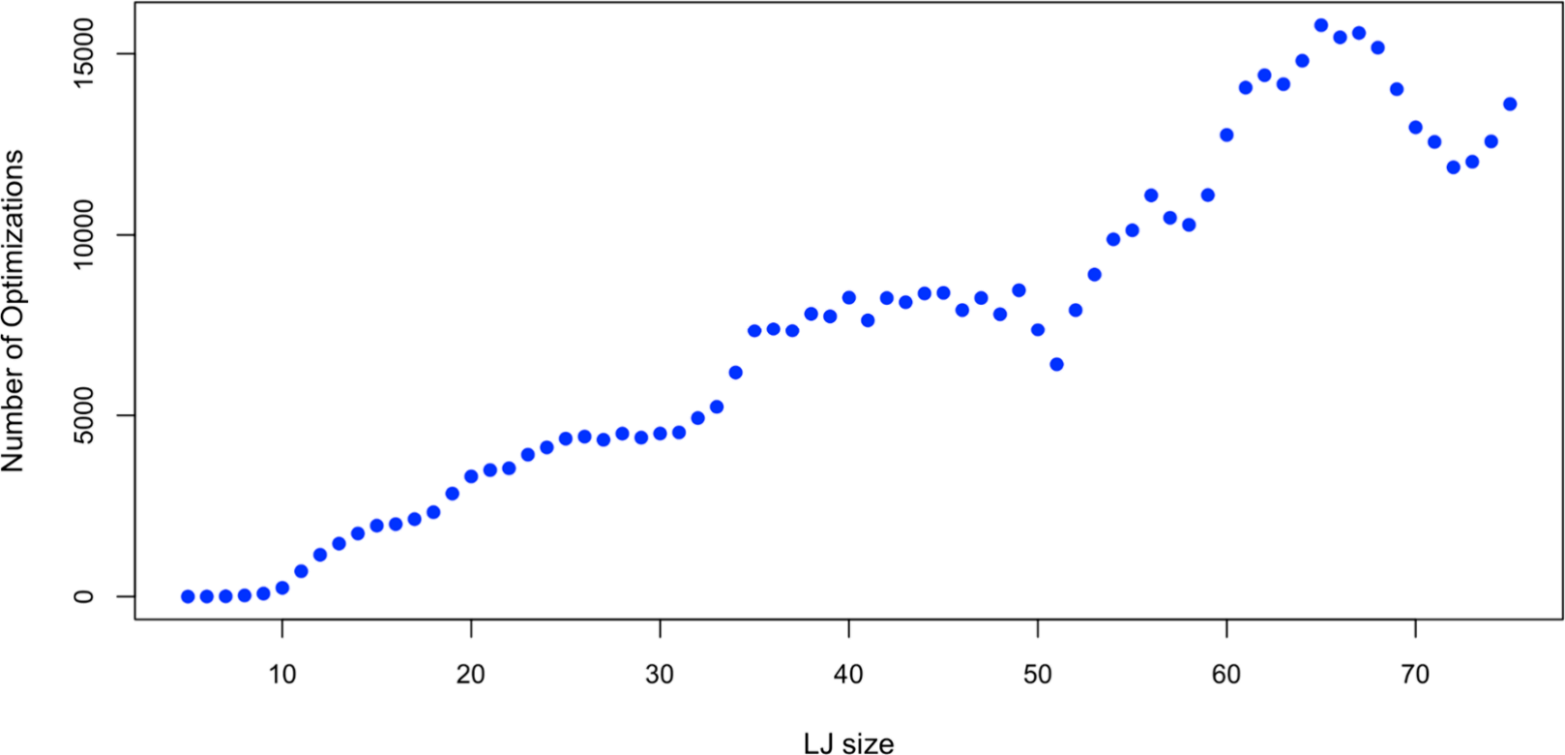
Independent tests (10 conducted for reliability on GrowPAL). Every test used 63 seeds to find the global minimum up to LJ_74_. The remaining 9 tests showed no discrepancies with this graph.

To further verify the reliability of GrowPAL, ten independent tests were conducted using identical initial seeds (LJ_4_) and parameters, with *N*_*max*_ set to 63 for each test. Test results show no variation in the number of optimizations and global minima identified (Figure 3). This consistency across different tests suggests that GrowPAL operates with a high level of determinism.

Although GrowPAL could not find the correct structures for LJ_75–78_, it identified isomers with energies of 1.2, 0.51, 0.57, and 0.1 eV, which were higher than the putative global minima. These are significant results, given the difficulty in determining the minimum for LJ_75–78_–a challenge that has puzzled even more sophisticated and robust unbiased algorithms in the past. For instance, AUTOMATON,^27,28^ which combines probabilistic cellular automata with a genetic algorithm, succeeded in locating the global minimum for LJ_38_ but failed for LJ_75_. The isomer found by AUTOMATON for LJ_75_ was significantly higher in energy (by 6.4 eV) than the reported global minimum, in contrast to the isomer found by GrowPAL, which was only 1.2 eV off. The Firefly algorithm,^29^ a stochastic optimization method inspired by the behavior and flashing patterns of fireflies, identified every minimum of LJ clusters with up to 63 atoms but could not find LJ_75_, even after 47,000 optimizations. Similarly, the Sophisticated Fast Annealing Evolutionary Algorithm (SFAEA),^30^ which uses seeding, similarity checking, and moving techniques, found all LJ minima up to 74 atoms but was unable to find LJ_75_ and required 118,146 energy evaluations to ascertain LJ_38_.

**Figure 4:**
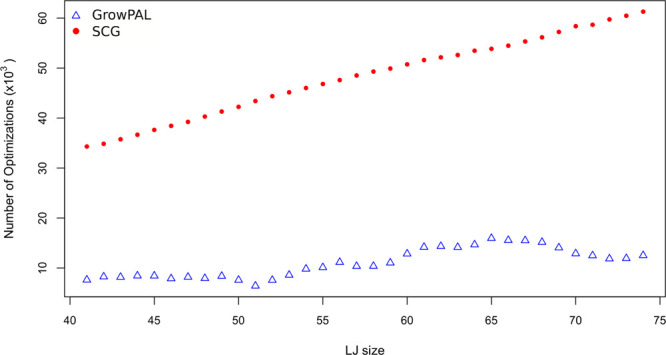
Comparison of total optimizations required by SCG and GrowPAL. SCG requires roughly four times the number of optimizations compared to GrowPAL to find the reported minimum.

Since GrowPAL is a biased algorithm, it is most suitable for comparing similar methods. To our knowledge, SCG^18^ is the only other algorithm that has performed a growth-based exploration for LJ clusters up to 80 atoms. In contrast to GrowPAL, SCG uses three distinct types of atom additions, substantially increasing the number of optimizations required. This discrepancy is evident in the case of identifying LJ_41_, where SCG requires 34,305 optimizations—more than four times the number of GrowPAL seeds (7,632). This trend of increased efficiency with GrowPAL over SCG is consistent for all LJ clusters evaluated up to 74 atoms (see Figure 4).

### Deconstruction Scheme

The challenge of finding the global minimum of LJ_75_ motivated us to perform an in-depth analysis to identify the obstacles GrowPAL faced. To this end, we introduced the Deconstruction Scheme (DS), which sequentially removes one atom from a known minimum structure, denoted as rLJ_*n*_, to generate *n* deconstructed structures, each labeled dLJ_*n-1*_, containing *n-1* atoms. These deconstructed structures are then relaxed and used as starting points for GrowPAL. If GrowPAL successfully reconstructs the original rLJ_*n*_ from these deconstructed forms, the process is repeated with further atom removals to find a set of precursor structures, or “forebears”, possessing the essential topological properties needed to assemble the target rLJ_*n*_. In this way, DS explores the “genealogical branch” or “lineage” of rLJ_*n*_ by detecting smaller forebear structures that are elusive to GrowPAL.

The exploration began with the deconstruction of rLJ_75_, where it was found that the optimal dLJ_74_ structure is 0.56 eV higher in energy than the corresponding rLJ_74_. Using these dLJ_74_ seeds, GrowPAL was able to locate the reported minimum for rLJ_75_. Further deconstructing the forebear dLJ_73_ and using these seeds allowed GrowPAL to identify dLJ_74_ and rLJ_75_. Intriguingly, the most favorable dLJ_73_ was 1.58 eV higher in energy than rLJ_73_. This suggests that certain forebear seeds remain elusive for GrowPAL, mainly due to their specific placements in the energy landscape and their respective symmetry.

Further explorations (Table 2) showed discrepancies between all other deconstructed dLJ_*n*_ structures and their corresponding minima for values up to *n* = 50, with dLJ_55_ displaying the most substantial differences of 9.32 eV from its global minimum. In contrast, during the initial execution of GrowPAL, the most energetic isomer found for LJ_55_ (ranked as the 63rd isomer) was only 5.20 eV higher than the reported minimum. These findings highlighted the limitations of our initial selection of 63 seeds. Although this number proved adequate for locating minima for clusters up to 74 atoms, it became clear that to describe rLJ_75_ accurately, it was necessary to augment the pool of seeds.

**Table 2:** Energetic Comparison between Isomers Obtained through the Deconstruction Scheme and Their Reported Counterparts^a^

**Cluster Size(n)**	**E (dLJ**_***n***_**)**	**E (rLJ**_***n***_**)**	**ΔE**
74	–390.351627	–390.908500	0.56
73	–383.210331	–384.789377	1.58
72	–375.695787	–378.637253	2.94
71	–369.127821	–373.349661	4.22
70	–362.812152	–366.892251	4.08
69	–357.500251	–359.882565	2.38
68	–350.149095	–353.394542	3.25
67	–343.713801	–347.252007	3.54
66	–337.440366	–341.110599	3.67
65	–332.147235	–334.971532	2.82
64	–325.873754	–329.620147	3.75
63	–319.548120	–323.489734	3.94
62	–313.590503	–317.353901	3.76
61	–306.254369	–312.008896	5.75
60	–299.877523	–305.875475	6.00
59	–293.523982	–299.738069	6.21
58	–287.327305	–294.378147	7.05
57	–282.061080	–288.342624	6.28
*56	–275.801093	–283.643105	7.84
*55	–269.927897	–279.248470	9.32
54	–263.779829	–272.208631	8.43
*53	–257.657508	–265.203016	7.55
52	–252.216995	–258.229991	6.01
51	–245.903039	–251.253964	5.35
*50	–240.568897	–244.549926	3.98

a The total energy for the best-deconstructed structure (dLJ_*n*_) and the reported global minimum (rLJ_*n*_) are shown, along with the energy gap (ΔE) between them. All energy values are in eV. An asterisk indicates the isomers at which the genealogical branch diverges towards a different one.

**Figure 5:**
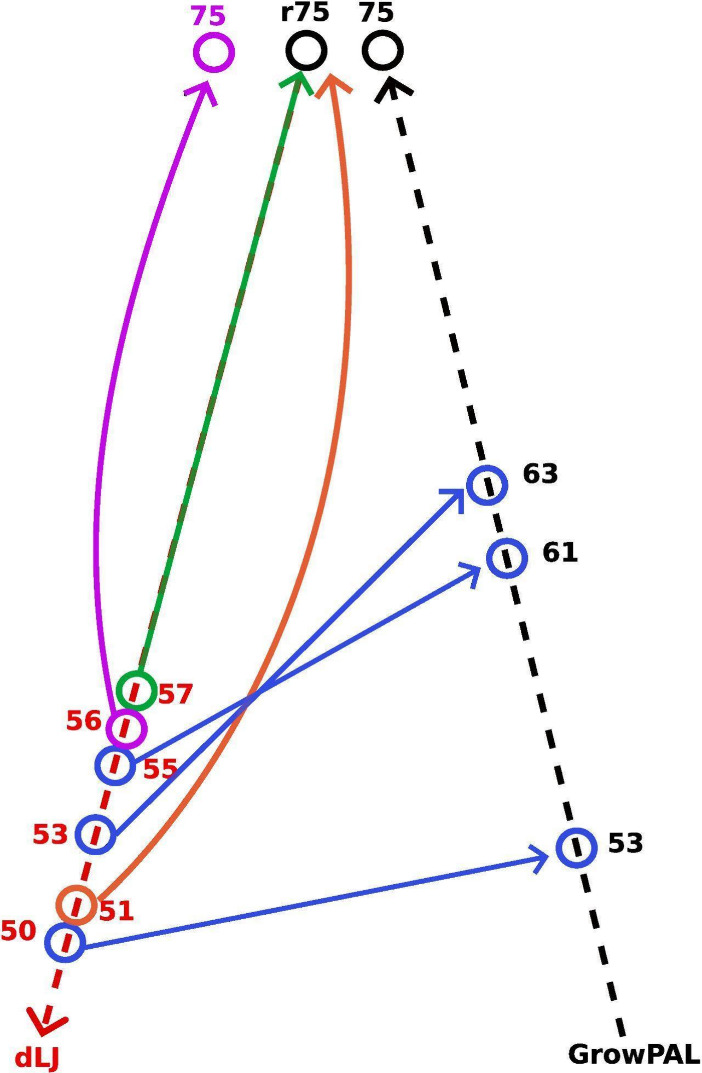
Growth branches in the Deconstruction Scheme (DS). Each dot along the dashed lines represents LJ clusters. The left line shows dLJ_*n*_ found by DS, while the right line shows rLJ_*n*_ found by GrowPAL. The green line indicates the main genealogical branch. Blue lines denote minor branches that connect dLJ_*n*_ with rLJ_*n*_ during growth from structures marked with circles. Orange and purple lines depict pathways diverging from the main lineage toward rLJ_75_ and LJ_75_, respectively.

The genealogical branch encountered a divergence at the deconstruction stage of dLJ_56_. The isomer of LJ_75_ resulting from this growth path exhibited a difference of 1.73 eV from the GrowPAL-identified structure and was 2.93 eV away from the global minimum. This instance marked a point where growth from forebear structures did not suffice to accurately identify rLJ_75_– a trend that persisted with further attempts. Growth processes initiated from deconstructed structures with 55, 53, and 51 atoms consistently led back to the LJ_75_ isomer in GrowPAL’s original execution. Intriguingly, initiating growth from dLJ_52_ exposed a new pathway that directly led to rLJ_75_. These findings highlight the existence of various growth pathways that converge and diverge, complicating the process of pinpointing the correct trajectory toward the global minimum. The success of GrowPAL, therefore, depends not just on the number of initial seeds but critically on their inherent quality. Figure 5 depicts these genealogical branches, as revealed through the DS, offering insight into the complex network of potential growth routes that strengthen the algorithm’s exploration challenges.

Given the energy discrepancies between the deconstructed forebear structures and reported seeds, coupled with the role of initial seed selection, we hypothesized that expanding the seed pool could address the challenge of failing to locate rLJ_75_. We adjusted the algorithm’s parameters to uncover the elusive structures and embarked on a test starting from LJ_10_, employing *N*_*max*_ = 1,500 seeds. While this strategy required more optimizations and extended processing time, it presented a promising avenue to capture the missing forebears. Our attention shifted upon finding dLJ_57_, marking the genesis of the genealogical branch under scrutiny. Note that the energy gap between dLJ_57_ and its reported counterpart, rLJ_57_, stands at 6.38 eV, indicating that any deviation from this energy threshold in the most energetic isomer using 1,500 seeds would imply the algorithm’s inability to locate the forebear seeds. However, the 1500th isomer is only 3.26 eV higher in energy, which is nearly half of the required energy gap. Addressing this situation may require a refined approach to seed selection that aligns with the *N*_*max*_ value. While potential solutions are being considered, their implementation remains pending.

The essence of global optimization strategies lies in their capacity to systematically explore a PES. In contrast, GrowPAL is designed to provide high-quality seeds, reduce search expenditures, and improve scalability. Our objective, therefore, is not to bypass these existing algorithms but to enhance their utility through complementary means.

### Sutton-Chen Clusters

Having evaluated the strengths and weaknesses of GrowPAL, we employed it to explore, we explored the PESs of clusters with 5 to 80 atoms described by the Sutton-Chen (SC) family of potentials.^31^ SC potentials are popular due to their adaptable computational form and ability to describe delocalized metallic bonding through an approximate many-body representation, providing agreement with various experimental situations. Doye and Wales identified the global minimum for the SC clusters 9–6, 12–6, and 10–8, incorporating them in the Cambridge Cluster Database (CCD), and used them as benchmarks for evaluating several global optimization algorithms.^32^ For a direct comparison, we used the n, m, a, c, and ε parameters provided by SC^31^ for Cu (*n* = 9, *m* = 6), Ag (*n* = 12, *m* = 6), and Au (*n* = 10, *m* = 8). Again, all structural optimizations were conducted using the GULP code, and each search began with SC_4_ structures provided by the genetic algorithm implemented in GLOMOS. For each case, *N*_max_ was set to 1000 to increase the probability of finding the correct genealogical branches. The results of each corresponding exploration are presented in Table 3.

**Table 3:**
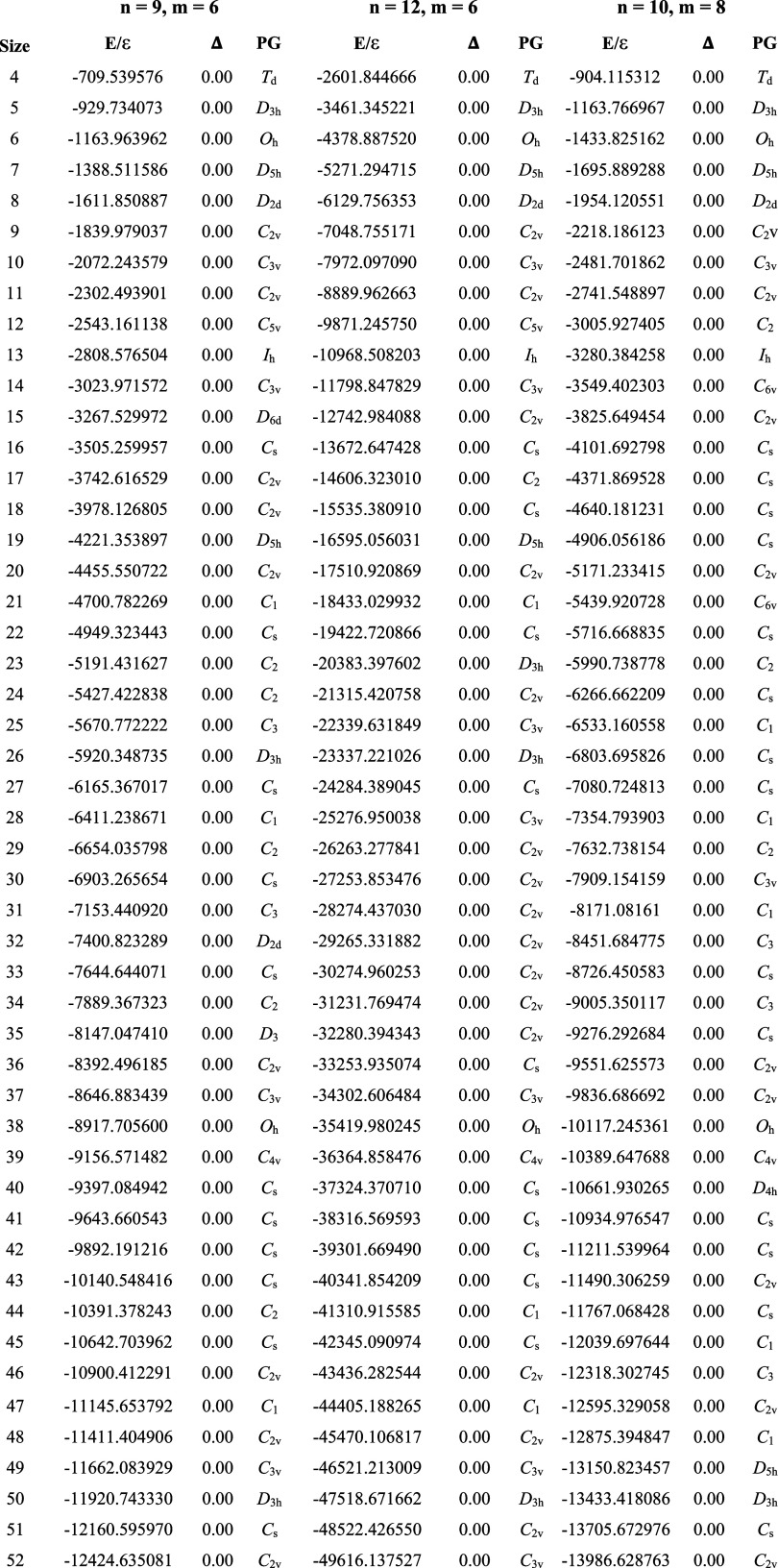

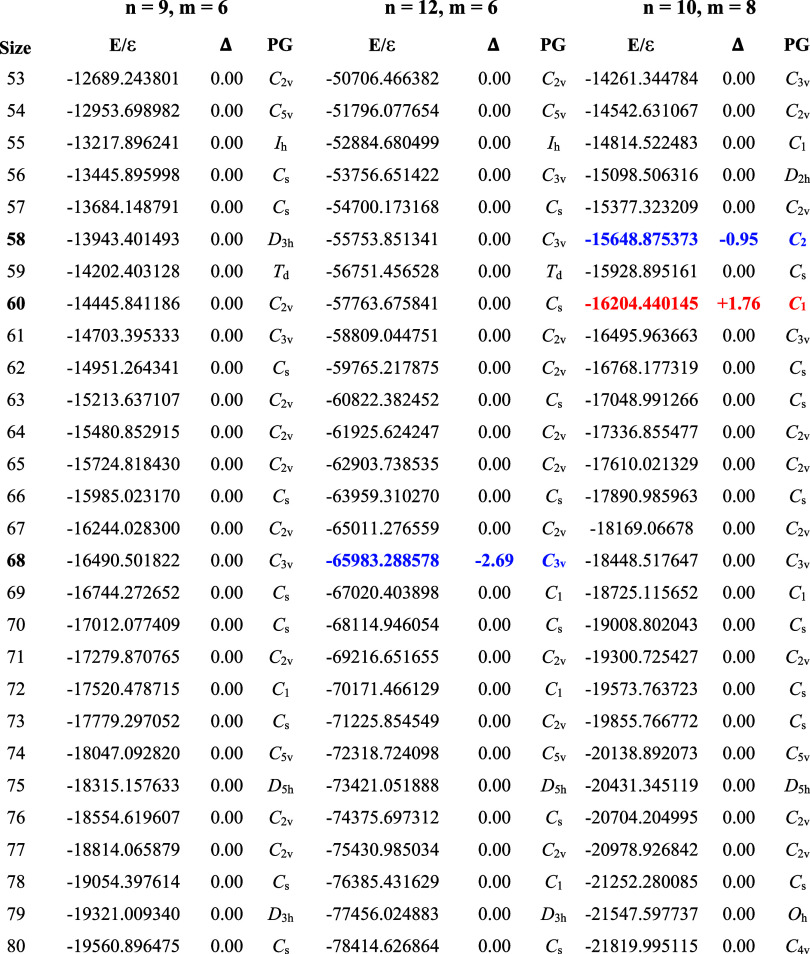
Sutton-Chen Clusters Found by GrowPAL (*N*_max_ = 1000)^a^

a E is the total energy, while Δ is the E/ε difference related to the minima in the CCD. GrowPAL failed to find the minimum for SC_60_ (*n* = 10, *m* = 8) but located new minima for SC_68_ (*n* = 12, *m* = 6) and SC_58_ (*n* = 10, *m* = 8). These cases are highlighted in bold, and a minus sign corresponds to the new minima, while a plus sign is for the one structure not found.

**Figure 6:**
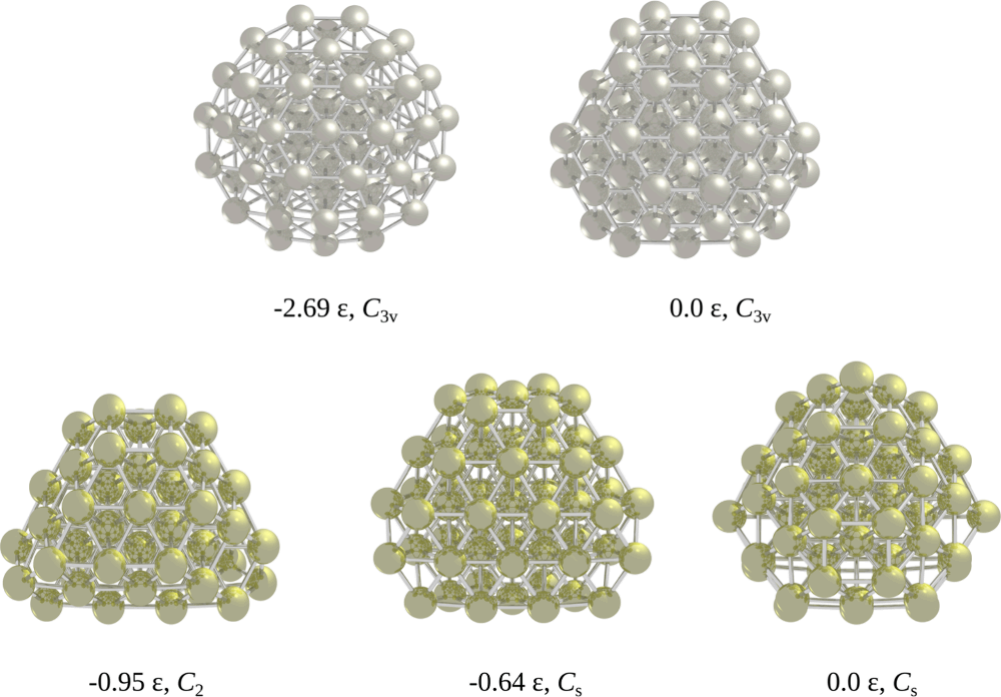
Top row shows the lowest energy structure found for SC_68_ (*n* = 12, *m* = 6), represented with Ag atoms in silver color. This new isomer is lower in energy by 2.69 ε from the previously reported one. The bottom row shows two new lowest energy isomers for SC_58_ (*n* = 10, *m* = 8), represented with Au atoms in gold color. The new global minimum is 0.95 ε under the reported in the CCD. The point group of each isomer is also shown.

Nearly every structure found by GrowPAL aligns with the findings reported by Doye and Wales in the CCD. The first noteworthy discrepancy is observed for SC_68_ (*n* = 12, *m* = 6), where a new minimum is identified! This structure lies 2.69 ε lower in energy than the reported structure and shares the same point group, *C*_3v_. Furthermore, we found two new low-energy isomers for SC_58_ (*n* = 10, *m* = 8). The lowest energy isomer, adopting *C*_2_ symmetry, lies at 0.95 ε below the reported one, contrasting with the reported structure with *C*_*s*_ symmetry. The second lowest energy structure exhibits an energy gap of 0.64 ε compared to the *C*_*s*_ form. However, GrowPAL failed to identify the current global minimum for SC_60_ (*n* = 10, *m* = 8), locating a *C*_1_ structure 1.76 ε higher. These results show the algorithm offers a high benefit ratio between success and computational cost. Figure 6 depicts the new low-lying energy isomers compared to the current CCD global minima structures, referenced by 0.0 ε. The Supporting Information includes the Cartesian coordinates for the new minima.

### Boron Clusters

We also applied GrowPAL to study the challenging case of boron clusters. Experimental and theoretical studies have indicated that small boron clusters tend to be planar. In 2005, the group of Wang provided experimental evidence of a 2D-to-3D structural transition in B_20_.^33^ However, in 2012, Cheng reported that B_14_ forms a small boron fullerene,^34^ marking it as the first instance of a 2D-to-3D transition in boron clusters. Initial studies of B_14_ suggested it to be planar, aligning with trends observed in smaller clusters. Therefore, the transition observed in B_14_ is a rigorous test for GrowPAL, particularly considering that numerous methodologies propose the planar structure of this cluster.

Drawing the insights gained through the Deconstruction Scheme, which underscored the importance of diversity in the initial seed pool, we initiated the search using ten of the top isomers of B_7_ identified by the Genetic algorithm in GLOMOS, including the global minimum. We selected this set of structures because both 3D and 2D structures were among the most energetically favorable. Note that if all forebears were exclusively two-dimensional, the lineage would likely consist solely of two-dimensional descendants. Therefore, it is mandatory to include both 2D and 3D structures among the initial seeds.

**Figure 7:**
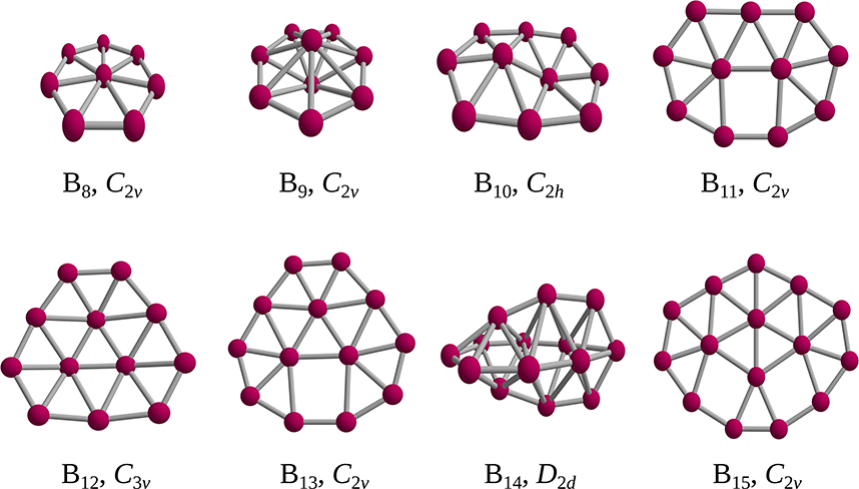
Minima found by GrowPAL for B_8–15_. Each structure shows its corresponding point group.

Regarding the results, we successfully calculated up to B_15_ using Gaussian16 at the ωB97XD/def2-SVP level. Each search used 20 of the top previous structures, yielding highly favorable outcomes as GrowPAL identified every global minimum documented in the literature. It is noteworthy that GrowPAL located the tubular structure reported for B_14_.^33^ All resulting structures are depicted in Figure 7.

## Conclusions

GrowPAL introduces an efficient methodology for finding global minima. We explored the energy landscape of Lennard-Jones clusters up to 80 atoms. Our findings include the 38-atom octahedron cluster and the 69-atom icosahedral structure with an incomplete core. One of the innovations behind GrowPAL’s success is adopting a new type of atom addition, the interstitial type, which is more effective than previously reported methods. Also, the algorithm successfully described every Sutton-Chen cluster for Cu (*n* = 9, *m* = 6), Ag (*n* = 12, *m* = 6), and Au (*n* = 10, *m* = 8) containing 5 to 80 atoms. GrowPAL could find a new global minimum for SC_68_ (*n* = 12, *m* = 6) and two new lowest energy configurations for SC_58_ (*n* = 10, *m* = 8). However, it failed to locate the reported minimum for SC_60_ (*n* = 10, *m* = 8). Moreover, GrowPAL was able to locate every minimum for boron clusters with 8 to 15 atoms, including the three-dimensional structure for B_14_. However, the Deconstruction Scheme revealed some of the methodology’s limitations, particularly its reliance on the quality of initial seeds. DS also showed that certain structures, which are crucial for achieving global minima through growth, may be energetically less favorable. This suggests that using a limited selection of initial seeds may not be sufficient to capture complex cases like LJ_75_. It is important to note that GrowPAL’s goal is not to provide the best solution but to generate valuable structures that can be used to improve the results of more advanced algorithms. This approach emphasizes GrowPAL’s usefulness in enhancing the search for global minima by efficiently exploring the potential energy landscape with minimal resource consumption.

## Supporting Information Available

Cartesian coordinates for the new low-lying energy isomers found for Sutton-Chen clusters with 58 (*n* = 10, *m* = 8) and 68 atoms (*n* = 12, *m* = 6) (PDF)
