# Supplementary material for: An Efficient Growth Pattern Algorithm (GrowPAL) for Cluster Structure Prediction
Source: J Chem Theory Comput. 2024 May 31;20(11):4939–48. doi: 10.1021/acs.jctc.4c00365 (PMC11171261; doi:10.1021/acs.jctc.4c00365)
Supplement: Supplementary file 1 — ct4c00365_si_001.pdf [file ct4c00365_si_001.pdf]

Supporting Information

An Efficient Growth Pattern Algorithm (GrowPAL) for Cluster Structure Prediction

Carlos López-Castro,[1] Filiberto Ortiz-Chi,[2,*] and Gabriel Merino.[1,*]

[1] Departamento de Física Aplicada, Centro de Investigación y de Estudios Avanzados del Instituto Politécnico Nacional, Mérida, 97310, Yucatán, México.

[2] Conahcyt-Departamento de Física Aplicada, Centro de Investigación y de Estudios Avanzados del Instituto Politécnico Nacional, Antigua Carretera a Progreso km 6, Mérida, Yucatán, 97310, México.

e-mail: fortiz@conahcyt.mx

gmerino@cinvestav.mx

Structure in Cartesian coordinates for the low-lying energy isomers for Sutton-Chen 10-8 clusters with N = 58. The new low-lying energy structures correspond to **SC-58[a]** and **SC-58b**, while **SC-58c** is the previously reported global minimum. The unit of distance is the lattice constant, a. The first line contains the values of the parameters in the Sutton-Chen potential ε, c, a, n, and m. The format of the structures followed was agreed upon in the Cambridge Cluster Database for the Sutton-Chen clusters (http://doye.chem.ox.ac.uk/jon/structures/SC/10-8/table.10-8.html).

SC-58[a]

```
1.0000000000   34.4080000000   1.0000000000   10.000000000   8.0000000000
  -0.3338707304   -0.5014902881    1.1243772109
   0.0081744868   -0.7257923817    0.5706696974
   1.0435379672    0.3035687754    0.0100935131
  -0.0002819576    0.0918215507   -1.1549918156
   0.3475277650   -0.9129084707    0.0102962310
  -0.3347720536    0.6823619364    1.1110301592
   0.6928817388   -0.6957110020    0.5630847209
  -0.3474153509   -0.9129515785   -0.0102954082
  -0.6817830975    0.1028738460    1.1234896633
```

0.3443393272	-0.1070289829	0.5650372604
-1.0435763909	0.3034393080	-0.0100926903
0.0002697824	0.0918214676	1.1549926383
1.0301111559	-0.8846508525	0.0101487550
-0.6927962546	-0.6957969016	-0.5630838979
0.3401768613	0.6759248254	1.1118882538
0.6772085412	0.0936870909	1.1094896629
1.3341109392	-0.6725699829	-0.5360913587
-1.0294260164	-0.1045089594	-0.5621960403
0.3368015547	1.0740647896	-0.5655119413
0.9962602427	-0.4796996856	-1.0791217994
-0.0012397818	0.4892875428	0.5637204200
-0.0000577208	0.9246195678	0.0000004116
0.3565610976	0.3043473607	-0.0025378322
0.6792702665	0.8953817041	0.0017488518
0.3398865964	1.0690924200	0.5673060278
-0.7079996279	0.5134096146	0.5678263570
0.3434898804	-0.4923357886	1.1107627136
-1.0538163105	-0.0732143395	0.5597240660
0.0000195138	-0.3211001655	0.0000004116
-0.3402614627	0.6758827291	-1.1118874310
-0.3565996233	0.3043031215	0.0025386553
-0.6793820777	0.8952974239	-0.0017480288
-0.3369355252	1.0740229389	0.5655127643
1.0294381785	-0.1043813003	0.5621968633
0.7113448142	0.5044871208	0.5702972285
-0.3561081152	-0.1121695332	0.5764869455
-1.3641969669	-0.3011475270	-0.0098849514
-0.6963519853	-0.3056708354	0.0065546239
-0.6772209424	0.0936031852	-1.1094888398
-0.3434296174	-0.4923782868	-1.1107618908
0.3561212321	-0.1121252932	-0.5764861224
1.3642335098	-0.3009782773	0.0098857744
-0.3400199505	1.0690503016	-0.5673052048
-0.0080852777	-0.7257933324	-0.5706688744
-0.3443268421	-0.1070716449	-0.5650364374
0.3339550098	-0.5014430837	-1.1244002536
0.3346866488	0.6824035774	-1.1110293364
-0.7114081544	0.5043989220	-0.5702964053
0.0011783209	0.4892877499	-0.5637195972
0.6817695466	0.1029585443	-1.1234888401
0.7079351706	0.5134975060	-0.5678255340
0.6963890937	-0.3055844400	-0.0065538011
0.6743332802	-0.7329435743	-0.5596512678
1.0538245917	-0.0730835410	-0.5597232433
-1.3340283193	-0.6727355479	0.5360921815

-0.6742431794	-0.7330272864	0.5596520906
-1.0300022404	-0.8847786449	-0.0101479320
-0.9962015414	-0.4798233922	1.0791226224

SC-58[b]

1.0000000000	34.4080000000	1.0000000000	10.000000000	8.0000000000
-0.3373655694	1.0408352899	0.7195261658		
0.0000280605	0.4667477774	0.8372875776		
1.0437787311	0.1266137921	-0.2842504969		
-0.0000103846	-1.0586050447	-0.3128477305		
0.3479620375	-0.1187312567	0.9059989410		
-0.3386470704	1.2797275850	-0.4247073065		
0.6900075164	0.4650750673	0.8070958734		
-0.3479828066	-0.1187129422	0.9059989410		
-0.6763098155	1.1589383855	0.1456199516		
0.3468215012	0.5838374794	0.2305283844		
-1.0437995003	0.1266137921	-0.2842504969		
0.0000344679	1.2033262492	0.1565288844		
1.0314827274	-0.1127302567	0.8782620334		
-1.3381090606	-0.6049160255	0.5608820663		
0.3386263012	1.2797336896	-0.4247245131		
0.6763595289	1.1589628046	0.1455798031		
1.3380882914	-0.6049221305	0.5608820663		
-1.0019008194	-1.0995009733	0.2624694180		
0.3374921728	-0.2817167032	-1.1530305521		
1.0018800502	-1.0995009733	0.2624694180		
-0.0000103846	0.7042481825	-0.3556231352		
-0.0000103846	0.2427816722	-0.8943764325		
0.3567082828	0.1126765769	-0.2881219636		
0.6794287242	0.2386365259	-0.8651482930		
0.3395105372	0.8275586563	-0.9214308148		
-0.7114678003	0.7157312757	-0.3672203286		
0.3377356581	1.0408291850	0.7195261658		
-1.3655203706	0.0041701970	0.3066959058		
-0.0000103846	-0.0178985868	0.3226979678		
-0.3367376336	-0.8959065229	-0.8807431342		
-0.3567290520	0.1126765769	-0.2881276992		
-0.6794494934	0.2386365259	-0.8651482930		
-0.3395313064	0.8275586563	-0.9214250793		
1.0301884113	0.5839473656	0.2282456531		
0.7114406235	0.7157251710	-0.3672203286		
-0.3468422704	0.5838252698	0.2305283844		
-0.6899642106	0.4651116960	0.8070671959		
-0.6969804294	-0.0144554903	0.3082330214		
-0.6831594392	-1.0254316640	-0.3158703423		

-0.3400759444	-1.1485773089	0.2741469084
0.3538120902	-0.5341921363	0.0035285229
1.3654996014	0.0041701970	0.3066959058
-0.3375129419	-0.2817167032	-1.1530305521
-0.0000103846	-0.6490474883	0.6070700972
-0.3538328593	-0.5341921363	0.0035285229
0.3400551752	-1.1485773089	0.2741469084
0.3367232720	-0.8959065229	-0.8807431342
-0.7085331625	-0.3974938773	-0.6056109053
-0.0000103846	-0.4006744682	-0.5800936386
0.6831386701	-1.0254316640	-0.3158703423
0.7085059858	-0.3974938773	-0.6056109053
0.6969596603	-0.0144554903	0.3082330214
0.6789801988	-0.6413371498	0.6144689001
1.0537936630	-0.5082346069	-0.0298463871
-1.0314970890	-0.1127119425	0.8782562979
-1.0538144321	-0.5082346069	-0.0298463871
-0.6790009679	-0.6413371498	0.6144689001
-1.0302155878	0.5839473656	0.2282513886

SC-58[c]

1.0000000000	34.4080000000	1.0000000000	10.000000000	8.0000000000
-0.0000000000	-0.1492999472	-0.3820587150		
0.6766154890	-0.1364647312	-0.3463039917		
0.3447223051	0.4854179670	-0.2920126422		
0.3453637613	0.1755622918	-0.9098863317		
0.3455479417	-0.7289253570	-0.6058108226		
0.3460877811	-0.5981592067	0.0714620343		
0.3486536054	0.0698444107	0.2578261049		
-0.3486536054	0.0698444107	0.2578261049		
-0.3460877811	-0.5981592067	0.0714620343		
-0.3455479417	-0.7289253570	-0.6058108226		
-0.3453637613	0.1755622918	-0.9098863317		
-0.3447223051	0.4854179670	-0.2920126422		
-0.6766154890	-0.1364647312	-0.3463039917		
1.0330237265	0.4864134273	-0.2778236000		
1.0303372324	0.1480627016	-0.8494240255		
1.0293528196	-0.6694714920	-0.5724793432		
1.0335572147	-0.5921242288	0.0869339701		
1.0270727924	0.0652404068	0.2533122196		
0.7038424414	0.6753891173	0.2944301512		
0.6757009377	1.0744255898	-0.2481685613		
0.6932361875	0.7356766800	-0.8134851834		
0.6843066108	-0.3755867325	-1.0519074184		
0.6929567412	-1.1148341972	-0.1862036064		

0.6731668686	-1.0398262656	0.4700975017
0.7043124191	-0.3953341756	0.6565209654
0.6792321216	0.2564372173	0.8281555157
-0.0000000000	0.6793274070	0.2888353093
-0.0000000000	1.1059630156	-0.2608905907
-0.0000000000	0.7574772602	-0.8283572476
-0.0000000000	-0.3809622180	-1.0676644417
-0.0000000000	-1.1407721589	-0.1878963135
-0.0000000000	-1.0746922616	0.4747955058
-0.0000000000	-0.3964540685	0.6407876992
-0.0000000000	0.2703674396	0.8556308228
-0.6792321216	0.2564372173	0.8281555157
-0.7043124191	-0.3953341756	0.6565209654
-0.6731668686	-1.0398262656	0.4700975017
-0.6929567412	-1.1148341972	-0.1862036064
-0.6843066108	-0.3755867325	-1.0519074184
-0.6932361875	0.7356766800	-0.8134851834
-0.6757009377	1.0744255898	-0.2481685613
-0.7038424414	0.6753891173	0.2944301512
-1.0270727924	0.0652404068	0.2533122196
-1.0335572147	-0.5921242288	0.0869339701
-1.0293528196	-0.6694714920	-0.5724793432
-1.0303372324	0.1480627016	-0.8494240255
-1.0330237265	0.4864134273	-0.2778236000
-1.3372898417	-0.1276362430	-0.3167024069
0.3496951775	0.8543542097	0.8508437286
0.3378123625	-0.2053443606	1.1951878463
-0.3496951775	0.8543542097	0.8508437286
-0.3378123625	-0.2053443606	1.1951878463
0.3464624929	1.2660205844	0.3038499171
0.3361483873	-0.8468065182	1.0150303679
-0.3464624929	1.2660205844	0.3038499171
-0.3361483873	-0.8468065182	1.0150303679
1.3372898417	-0.1276362430	-0.3167024069
-0.0000000000	1.4043833411	0.8399331926

Structure in Cartesian coordinates for the low-lying energy isomers for Sutton-Chen 12-6 clusters with N = 68. The new putative global minimum corresponds to **SC-68**[a], while **SC-68b** is the previously reported. The unit of distance is the lattice constant, a. The first line contains the values of the parameters in the Sutton-Chen potential ε, c, a, n, and m. The format of the structures followed was agreed upon in the Cambridge Cluster Database for the Sutton-Chen clusters (http://doye.chem.ox.ac.uk/jon/structures/SC/12-6/table.12-6.html).

SC-68[a]

1.00000000	144.41000000	1.00000000	12.00000000	6.00000000

0.6974960022	0.3914604246	0.2804832044
-0.3296142570	1.5209305432	0.0563972044
-0.6997754878	0.0074194285	0.8540688629
-0.0132148944	-1.0908653219	-0.3597541112
0.6042274135	0.3398111446	-0.9251558838
-0.0140957655	-1.2031186285	0.8430745492
1.1741666560	-0.0114868209	-0.7317352895
0.5601099773	-0.7349658522	-0.1116508511
0.5664929037	-0.3360712710	-0.7058849728
-1.1746594880	0.0166657285	-0.7308340939
0.5727240271	-1.0249622952	-0.7318948640
0.5616067888	-1.1469385324	0.4609244944
1.2757362359	0.0564414415	0.4611139741
0.3570810528	0.6021049278	0.8538956362
-0.3411358132	0.2024906706	-0.3040831330
0.6011058496	1.0091348857	-0.7311277332
0.9512389922	0.5343129283	-0.3594982220
-1.5128429220	0.2210311014	-0.1659662464
-0.9187910183	1.2220502777	-0.1658086337
-1.1523439511	-1.0459717304	0.0557274489
0.7004095714	-0.0093648011	0.8535316404
0.0156289736	1.3199342043	-0.5048426457
0.5649577998	-1.4205219529	-0.1673817027
-0.5966237929	0.3542028060	-0.9246951428
-0.5885755645	-1.1331542915	0.4613657949
1.1269836621	-1.0732892791	0.0548529191
0.3448702364	-0.2047137615	0.2861795352
-1.0341215306	0.6135607645	0.8441559807
0.9134955814	-0.1394898957	-0.1115570929
-0.3418905951	0.6104856762	0.8541638181
0.9476908998	1.1996832657	-0.1665247621
0.7125886548	1.0594167813	0.4617110242
0.3564248856	0.8526561011	-0.1109664882
0.9252565166	-0.5491561590	0.4645409328
-0.0086613729	-0.6929589781	-0.9253184980
1.0491782491	0.5885914459	0.8433566624
0.3436670665	-0.6105025458	0.8534369895
0.0003285254	-0.0003304121	0.8651853032
1.4820233890	-0.4750223681	0.0549471176
1.5175750963	0.1847160070	-0.1671289521
1.2907027010	0.7245758368	0.2124752658
-0.3575463445	-0.6020990646	0.8537060299
-0.0003417540	0.0003451730	-0.9033620655
0.0130706103	1.0754586351	0.4655080323
0.0076286083	0.6592629664	-0.7052924687
0.3660170237	1.5125935131	0.0561303054
0.3456615592	0.1942614591	-0.3043466418
-1.2728092174	0.7553072989	0.2134533761
-1.1510341980	-0.6459808796	-0.5051521846
-0.3359678792	0.8609541576	-0.1107008318
0.0049112181	0.4007666129	0.2865399631
-0.5773071619	1.0232614058	-0.7306755997

-0.5976879132	-1.0109296607	-0.7314457997
-0.6877168259	0.4080791104	0.2810092299
-0.6866339993	1.0761841500	0.4622478753
1.1348325117	-0.6733739377	-0.5060292222
-0.5989716694	-1.4065750673	-0.1669351288
-0.5776510354	-0.7213300045	-0.1112143164
-0.0048698631	-0.3964031475	-0.3044396420
-0.0094805584	-0.7998704490	0.2802901631
-1.4929355051	-0.4393677896	0.0560885462
-0.3494617934	-0.1963861947	0.2864459379
-1.2736621398	0.0869918542	0.4620921228
-0.9384308090	0.5569644654	-0.3587731941
-0.9166581961	-0.1175559915	-0.1108549019
-0.5749230440	-0.3223894840	-0.7054470349
-0.0176576252	-1.4801320133	0.2121226651
-0.9377952535	-0.5268286116	0.4652557460

SC-68[b]

1.00000000 144.41000000 1.00000000 12.00000000 6.00000000

0.0006870140	-0.4070328632	-0.4454582703
-0.3486279995	-0.2020653654	-1.0193082080
-0.3459481975	-1.0185970701	-0.4367281324
0.3502116367	-0.6062897033	0.1329924439
0.3483960032	-1.0054278977	0.7038468042
1.0442422172	0.2045024737	0.7038468042
0.0020427758	-1.2134181588	0.1372113145
0.7001672010	0.0022086947	0.1329924439
0.7081651076	-0.4072734156	0.7049805886
1.0498330729	0.6084770610	0.1372113145
0.3521557996	0.2041066079	-0.4454582703
-0.0006800586	0.4029536781	-1.0193082080
0.7064323084	0.8112788177	-0.4367281324
0.0000000964	-0.0000000909	0.1252349732
-0.3528425249	0.2029259827	-0.4454582703
-0.3481692836	-0.6074679581	0.1329924439
0.3499594262	0.6064368140	0.1329924439
-0.6938243423	-0.4021348857	-0.4329508410
0.0006754475	-0.4015711906	0.7012152316
0.3474353648	0.2013718429	0.7012152316
0.6965256829	0.8044372605	0.7038468042
-0.3450171973	-1.0065948657	0.7038468042
-0.0013488736	0.8019353732	-0.4329448735
0.6951735049	-0.3998007593	-0.4329508410
0.3493083470	-0.2008885852	-1.0193082080
1.0531970883	-0.6057035987	0.1409528023
0.3493722379	-1.0174282381	-0.4367281324
1.0551053736	0.2097014231	-0.4367281324
0.0013570426	-0.8075607058	-1.0061085733
0.6989764198	-1.1968788962	0.1364415348
1.3860298553	-0.0022305742	0.1364415348
0.6986916991	0.4049537387	-1.0061085733
-0.7001707738	-0.0001473828	0.1329924439

-0.0013721083	0.8169252668	0.7049805886
-0.3481105234	0.2001990756	0.7012152316
-0.3519976286	0.6052589915	0.1329924439
0.6959318137	-0.7983723517	-0.9940844938
1.0404840736	-0.9955287190	-0.4302297588
1.3837482541	-0.3986679632	-0.4302237916
1.0400525981	-0.2000133271	-0.9940844938
-1.0558042572	0.2061491480	-0.4367281324
-0.7091569968	0.8089016580	-0.4367281324
-1.0511533315	-0.6092419488	0.1409528023
-0.7067927102	-0.4096521234	0.7049805886
-0.7000484528	0.4026066950	-1.0061085733
-0.6949440569	-1.1992235224	0.1364415348
-1.0449278383	0.2009902524	0.7038468042
-1.0518691185	0.6049409380	0.1372113145
-1.3860186814	-0.0068892999	0.1364415348
-1.0393752628	-0.2035063476	-0.9940844938
-0.6932399545	-0.8007095136	-0.9940844938
-1.0371330650	-0.9990278454	-0.4302297588
-1.3823982144	-0.4033179488	-0.4302297588
-0.6992310628	0.8020857240	0.7038468042
-0.6910855090	1.2014538245	0.1364415348
-0.0020434677	1.2149452754	0.1409528023
0.6870425507	1.2037679238	0.1364415348
-0.3466149000	1.3976955363	-0.4302237916
0.3434436286	1.0018847104	-0.9940844938
0.3419143198	1.3988526791	-0.4302237916
-0.3468123547	1.0007225685	-0.9940844938
-0.6914203232	-0.0005288923	1.2632022064
-0.3472617669	0.5978810732	1.2632022064
0.0000000964	-0.0000000909	1.2804953986
0.3452494025	0.5990448671	1.2632022064
-0.3441491887	-0.5996795131	1.2632022064
0.3461648778	-0.5985226429	1.2632022064
0.6914176864	0.0017982800	1.2632022064
